# Corrigendum to “Historical flaws in bioassays used to generate metabolizable energy values for poultry feed formulation: a critical review”

**DOI:** 10.3382/ps/pez577

**Published:** 2019-09-23

**Authors:** Shu-Biao Wu, Mingan Choct, Gene Pesti

**Article ID: pez511**

Table 2 has been updated to remove first two rows showing r and P values of correlations for wheat vs wheat, and last two columns showing r and P of correlation for animal fat vs animal fat. All the r and P values between same ingredients are now removed where P values in the original Table were incorrect.

